# Executive Functions, Pragmatic Skills, and Mental Health in Children With Congenital Cytomegalovirus (CMV) Infection With Cochlear Implants: A Pilot Study

**DOI:** 10.3389/fpsyg.2019.02808

**Published:** 2020-01-10

**Authors:** Ulrika Löfkvist, Lena Anmyr, Cecilia Henricson, Eva Karltorp

**Affiliations:** ^1^Department of Special Needs Education, University of Oslo, Oslo, Norway; ^2^Department of Clinical Science, Intervention and Technology, Karolinska Institutet, Solna, Sweden; ^3^Karolinska University Hospital, Stockholm, Sweden; ^4^Department of Behavioural Sciences and Learning, Linköping University, Linköping, Sweden

**Keywords:** executive functions, pragmatcis, mental health, cytomegalo virus infection, cochlear implant

## Abstract

Congenital cytomegalovirus (cCMV) infection is the most common cause of progressive hearing impairment. In our previous study around 90% of children with a cCMV infection and CI had severely damaged balance functions (Karltorp et al., 2014). Around 20% had vision impairment, 15% were diagnosed with Autism-Spectrum-Disorder, and 20% with ADHD. One clinical observation was that children with cCMV infection had problems with executive functioning (EF), while controls with a genetic cause of deafness (Connexin 26 mutations; Cx26) did not have similar difficulties. A follow-up study was therefore initiated with the main objective to examine EF and pragmatic skills in relation to mental health in children with a cCMV infection and to draw a comparison with matched controls with Cx26 mutations (age, sex, hearing, non-verbal cognitive ability, vocabulary, and socioeconomic status level). Ten children with a cCMV infection and CI (4.8–12:9 years) and seven children with CI (4:8–12:8 years) participated in the study, which had a multidisciplinary approach. Executive functioning was assessed both with formal tests targeting working memory and attention, parent and teacher questionnaires, and a systematic observation by a blinded psychologist during one test situation. Pragmatics and mental health were investigated with parent and teacher reports. In addition, the early language outcome was considered in non-parametric correlation analyses examining the possible relationships between later EF skills, pragmatics, and mental health. Children with cCMV had a statistically significant worse pragmatic outcome and phonological working memory than controls despite their groups having similar non-verbal cognitive ability and vocabulary. However, there were no statistical differences between the groups regarding their EF skills in everyday settings and mental health. There were associations between early language outcomes and later EF skills and pragmatics in the whole sample.

**Conclusion:** Children with a cCMV infection are at risk of developing learning difficulties in school due to difficulties with phonological working memory and pragmatic skills in social interactions.

## Introduction

This explorative follow-up study is part of a larger research program with the objective to investigate the effects of different etiological backgrounds in children with pediatric deafness. We have investigated the effects of congenital cytomegalovirus (cCMV) infection in a sample of deaf children with cochlear implants (CI), and results have been related to their executive functioning, pragmatic skills, mental health, and possible interactions with the participants' early language outcome. This has been done in a group of children with CI, deafened due to cCMV infection, and in hearing-matched controls with a genetic cause of deafness: Connexin 26 mutations (Cx26). Congenital CMV infection is known to be related to comorbid conditions, while Cx26 is usually not related to other issues or diagnoses.

Executive functions (EF) are connected to frontal lobe capacity (Kave et al., 2008) and represent underlying, interrelated processing skills, such as working memory, attention, and inhibition/flexibility, which all are important for several functions like communication, social cognition, and learning (Miyake et al., 2000; Diamond, 2013). Children with CI form a heterogeneous population with considerable variation, especially in EF (Figueras et al., 2008; Beer et al., 2014; Kronenberger et al., 2014) but also in the spoken language outcome (Boons et al., 2012; Löfkvist et al., 2012; Walker et al., 2019) and mental health (Hintermair, 2007; Anmyr et al., 2012; Lingås-Haukedal et al., 2018). Poor EF in children with CI may negatively influence pragmatic skills, and especially in subgroups with known comorbid conditions like children with a cCMV infection. Poor attention skills and inferior ability to interpret and use pragmatic cues could affect emotional responses and behavioral actions in social interactions. In turn this might affect personal relationships and mental health. It is therefore valuable to explore the complex relationship between EF, pragmatic skills, and mental health.

Some of the language variation in the population of children with CI may be explained by age at implantation (Dettman et al., 2007; Colletti et al., 2011), non-verbal cognitive ability (Geers et al., 2011), parental sensitivity (Quittner et al., 2013), and socio-economic status (Szagun and Stumper, 2012). Phonological working memory is one EF ability that has previously been associated with language outcome (Gathercole et al., 2008; Wass, 2009), language learning (Willstedt-Svensson et al., 2004), and social interaction (Lyxell et al., 2008). Better language abilities may have positive effects on mental health (Lingås-Haukedal et al., 2018).

The cause of deafness and comorbidity could contribute to explaining some of the still unknown variations in cognitive processing, including poorer EF, which can negatively influence pragmatic skills and/or mental health in preschool and school-aged children with CI. Goberis et al. (2012) investigated pragmatic skills in children aged 3–7 years (*n* = 126) with hearing impairment (HI) and in controls with typical hearing (TH) (*n* = 109). They found that children with HI acquired pragmatic skills at a slower pace than controls with TH, even with targeted intervention strategies (Goberis et al., 2012). Goberis et al. (2012) did not investigate the possible effects of the cause of deafness in their study cohort.

Half of all sensorineural deafness (50%) is explained by genetic reasons (70% non-syndromic and 30% syndromic) (Alford et al., 2014). The most common non-syndromic genetic causes of deafness are Cx26 mutations (*GJB2*); they are manifested as congenital uni- or bilateral hearing loss/deafness, which can also be progressive. The other 50% of sensorineural deafness is acquired before birth, in infancy, or in early childhood and is explained by non-genetic causes like virus-infections, meningitis, or toxicity during pregnancy (Alford et al., 2014). Congenital cytomegalovirus (cCMV) infection is the most common cause within this group of congenital or early acquired hearing loss/deafness (Grosse et al., 2008). The heterogeneity and incidence of comorbid deficits or diagnoses are high in children with a cCMV infection compared to children with Cx26 deafness who usually do not have other additional diagnoses or deficits related to their cause of deafness (Karltorp et al., 2014).

Congenital CMV infection has a birth rate of 5% per 1,000 births. It has previously been suggested that 80% of all infants who are infected with a CMV infection *in utero* will develop typically without persisting deficits or difficulties within the area of perception, cognition (including language), or motor skills (Boppana et al., 2005). Around 15% of all children with a cCMV infection are diagnosed with a sensorineural HI (Boppana et al., 2005; Grosse et al., 2008). However, some of the children who are born with a cCMV infection will experience a late onset of their hearing loss and will thus not be identified through the universal newborn hearing screening (UNHS) system. Up to 40% of all infected children with cCMV will pass the Oto-Acoustic-Emission (OAE) test at the time when they are born (Fowler et al., 2017). Instead, they will experience later detection and diagnosis of their unilateral or bilateral HI, which may also be progressive (Fowler et al., 2017). So far, it is only the public health care system in the province of Ontario, Canada, that has decided to implement a general cCMV screening, as part of their existing UNHS system, for all newborns [(https://www.newbornscreening.on.ca/en/page/congenital-cytomegalovirus), retrieved 2019-11-24]. Several countries and states in the USA have started to screen for cCMV in all infants who are identified with a hearing loss through the UNHS. Aside from identifying a minority of all children with cCMV infection and HI, the UNHS system will only target infants with cCMV infection who have an HI and not the ones with initially TH but who might have other deficits and clinical symptoms. In the literature, there are reports of children who do not have HI but have vision impairments, motor skills deficits, balance problems, and/or cognitive deficits and, in some cases, neurodevelopmental diagnoses like mental retardation, cerebral palsy (CP), autism spectrum disorder (ASD), or ADHD, and this includes a negative impact on quality of life (Malm and Engman, 2007; Korndewal et al., 2017).

There are several studies that have investigated spoken language in relation to EF abilities like phonological working memory in children with CI (Lyxell et al., 2008; Beer et al., 2014; Kronenberger et al., 2014). Only a few studies have examined more general language abilities like the development of sentence understanding and speech intelligibility in children with a cCMV infection who use CI (Ramirez Inscoe and Nikolopoulos, 2004; Yoshida et al., 2009). Yoshida et al. (2009) found that language understanding developed at a slower pace in children with cCMV infection (*n* = 4) compared to children with CI who were deafened due to other causes. Ramirez Inscoe and Nikolopoulos (2004) showed in their study that there was a large variability concerning the speech intelligibility level in their cohort of 16 children with cCMV.

We have previously reported that some children with cCMV can catch up and develop adequate speech and language abilities over time, while others may have comorbid conditions (Karltorp et al., 2014). In the study by Karltorp et al. (2014), we found that children with cCMV, including those with typical language test results, had poorer impulse control and attention span during the language and hearing assessment procedure compared to controls with Cx26. On this test occasion we had no formal evaluation of EF, pragmatic skills, or mental health, and there was no psychologist involved in the research team (Karltorp et al., 2014). This unexpected finding was the first indication for us that EF, in particular, could be more difficult for children with cCMV.

Children with profound HI who use CI have been reported to have mental health issues more frequently than peers with TH (Hintermair, 2007). Nevertheless, recent findings in a Norwegian study displayed that the mental health of children with CI, aged 5; 0–12; 11 years, was similar to age-matched children with TH (Lingås-Haukedal et al., 2018). Lingås-Haukedal et al. (2018) examined health-related quality of life in 186 children with CI, as reported by parents, and they found that about 50% of the children with CI had levels comparable to peers with TH (*n* = 80). The possible influence on mental health in relation to the cause of deafness was not examined in the study by Lingås-Haukedal et al. (2018).

Mental health can be assessed with the Strengths and Difficulties Questionnaire (SDQ), which was originally developed in nearly identical versions for parents and teachers of children aged 4–16 (Goodman, 1997; Goodman et al., 1998). The SDQ can be used as part of a clinical assessment, as a treatment–outcome measure, and as a research tool (Goodman et al., 2000). The SDQ has been found to be a reliable and valid questionnaire for use in samples of deaf/hard of hearing children (Cornes, 2007; Hintermair, 2007). In a study by Hintermair (2013) EF and mental health were evaluated in children with HI, and in relation to their social communication skills by using two questionnaires—Behavior Rating Inventory Executive Function (BRIEF) (EF abilities; Gioia et al., 2000) and SDQ (mental health)—together with a communicative competence scale (Hintermair, 2013). The questionnaires were rated by teachers of 214 children with HI, who had a mean age of 12;4 years, and results were compared to normative data of 720 children. There was a statistically significant higher rate of EF difficulties in all children with HI compared to the norm data. Children who attended mainstream schools were rated to have better communicative competence than children who attended special schools for deaf children. A regression analysis revealed that better executive functioning and communicative competence in children with HI was associated with a lower incidence of behavioral problems (Hintermair, 2013). Seemingly, difficulties in verbal language abilities were not only related to EF outcome but also to social behavior in children with HI (Hintermair, 2013). Worse EF may have an influence on literacy, prosody, and language abilities (Lyxell et al., 2009) and may also negatively affect pragmatic skills in children with HI (Goberis et al., 2012; Hintermair, 2013), especially for children with initially atypical brain patterns in early childhood (Kave et al., 2008; Korndewal et al., 2017). Poor phonological working memory and short attention span are, for instance, known to affect children's ability to understand instructions and to retrieve words from their long-term memory (Lyxell et al., 2009). These difficulties can be negatively associated with linguistic and social skills in verbal interactions, in particular with regard to interactions in noisy environments, such as classrooms or playgrounds. To our knowledge, there are no previous studies in the literature that have explored cognitive abilities, like EF, pragmatic skills, as well as mental ill health in children with cCMV who use CI.

The objective of the present study was to explore EF, pragmatic skills, and mental ill health in children with an acquired deafness (cCMV infection) using CI and who have no known additional diagnoses like ADHD, Developmental Language Disorder (DLD), or Autism-Spectrum-Disorder (ASD) and compare this to well-matched controls who were deafened due to a genetic non-syndromic deafness (Cx26 mutations). The groups were matched on the basis of age, hearing, vocabulary, parents' education level, and non-verbal cognitive ability.

Several research questions were addressed:

1. Do children with CI have *worse EF* results in relation to norm data, regardless of the cause of deafness, and do children with cCMV infection have even poorer executive functioning compared to children with genetic non-syndromic deafness (Cx26)?
  *The hypothesis was that all children with CI would have a worse EF outcome than children with TH (norm data) (Kronenberger et al.*, *2014**), and that children with a cCMV infection would have even poorer EF results than children with Cx26 mutations. The reason for this hypothesis was that a congenital virus infection may be related to additional diagnoses, atypical brain patterns, and virus-related deficits (Karltorp et al.*, *2014**; Korndewal et al.*, *2017**)*.
2. Do children with a cCMV infection who use CI have *worse pragmatic skills and mental health* than well-matched children with Cx26 in comparison to norm data?
  *The hypothesis was that children with a cCMV infection would have worse pragmatic skills than controls. We hypothesized that worse pragmatic skills in children with cCMV infection may be explained not only by their deafness but also by the consequences of their congenital virus infection (Korndewal et al.*, *2017**)*.
3. Is there a relationship between EF, pragmatic skills, mental health, and early language abilities in children with CI, regardless of the cause of deafness?
  *The hypothesis was that there would be a relationship among EF, pragmatic skills, and mental health in all children with CI, regardless of their cause of deafness and that better speech and language understanding in early childhood could be related to an improved later outcome (Goberis et al.*, *2012**; Hintermair*, *2013**)*.

## Methods

The current follow-up study had a long-term approach, which included data collection and retrospective reviews of medical journals, and it is part of a larger research study program at the Auditory Implant Center, Karolinska Institutet, aiming to explore the effects of etiological factors in children with CI, who have different causes of deafness, in relation to their listening skills, cognitive abilities, mental health, and linguistic outcome. This study was carried out in accordance with the recommendations of the Regional Ethical Review Board in Stockholm, Sweden. All participants were first provided with written information about the study. Written informed consent was then obtained from the parents of all participants, in accordance with the Declaration of Helsinki. The protocol was approved by the Regional Ethical Review Board in Stockholm, Sweden; DN 2012:/2.

### Participants

Inclusion criteria: children with cCMV or Cx26 who were older than 4 years and younger than 13 years at the time of the study, who used their CI during all waking hours, who did not have a confirmed and known additional diagnose(s) related to deficits in the domain of executive functioning (ASD, ADHD) or pragmatic skills (DLD, ASD), and who had at least one parent who spoke Swedish at home. Families with a child who fulfilled the criteria and who had been implanted at the Auditory Implant Center, Karolinska University Hospital, which covers half of the Swedish population (i.e., five million people), were invited to take part in the follow-up study. Parents were first provided with written information about the study and then, if interested, they were asked to sign an informed consent of participation form. Children who could read (older than 8 years) also signed a consent of participation. Seven children with cCMV were excluded because they were too young or too old, and two children with cCMV were excluded because they had several additional diagnoses aside from their deafness. There was one participant with Cx26 who fulfilled the criteria and who initially agreed to participate but later decided not to participate in the study.

The final study sample consisted of 17 children (*N* = 17) aged 4.8–12.9 years (mean 8.2; Md 7; 8 years)—eight girls and nine boys with a confirmed cCMV infection or Cx26 mutations with CI who met the inclusion criteria. There were no statistically significant differences between the groups (cCMV vs. Cx26) regarding age (Z = −0.05, *p* = 0.96, *r* = 0.01), sex [χ^2^ (1, *n* = 17) = 1.63, *p* = 0.34] or parent educational level for mothers (Z = −0.40, *p* = 0.69, *r* = 0.10) or fathers (Z = −1.53, *p* = 0.13, *r* = 0.37). All parents had at least a high school or a university degree, which is common to most parents in the Swedish population. The participants came from different parts of Sweden, and a majority came from the Stockholm area. All but one family had been offered some kind of Family-Centered Early-Intervention (FCEI) option. Nine families had received regular services (once a week or every second week) from a speech–language pathologist or a teacher of the deaf at their local habilitation team using an auditory verbal approach for at least 1 year after the first CI surgery. Seven families had received similar intervention options but less frequently. One child, who was identified late with severe-to-profound hearing loss, had not received FCEI services with focus on parent engagement and spoken language skills before or after the first CI surgery (Table 1).

**Table 1:** Participant demographics concerning ages (months) when individual children were identified with a hearing impairment (HI), ages at identification of cause of deafness (cCMV or Cx26), ages when the children received their 1st and 2nd CI; type of Family-Centered Early Intervention (FCEI) actions after identification of HI, and the chronological ages of the children at the follow-up study.

**Child**	**Age at HI id**.	**Age at id. of etiology**	**Age at 1st CI**	**Age at 2nd CI**	**FCEI**	**Age at follow-up**
CMV-1	18	24	22	22	1	128 (10.7 year)
CMV-2	13	24	17	72	1	82 (6.8 year)
CMV-3	36	44	44	44	2	130 (11.0 year)
CMV-4	0	10	12	12	1	106 (8.2 year)
CMV-5	0	0	10	16	1	63 (5.3 year)
CMV-6	6	16	17	39	2	67 (5.6 year)
CMV-7	12	20	21	24	1	118 (9.8 year)
CMV-8	0	18	18	30	2	155 (12.9 year)
CMV-9	0	9	9	9	2	57 (4.8 year)
CMV-10	30	64	67	^*^	3	81 (6.8 year)
Md (min-max)	9 (0–36)	19 (0–64)	18 (9–67)	24 (9–72)	2 (1–3)	99 (57–155)
Cx26-11	2	10	8	49	1	108 (9.0 year)
Cx26-12	2	52	48	^*^	2	70 (6.0 year)
Cx26-13	10	19	19	23	2	153 (12.8 year)
Cx26-14	1	9	9	9	1	57 (4.8 year)
Cx26-15	19	23	22	27	2	140 (11.8 year)
Cx26-16	0	0	14	^*^	2	64 (5.3 year)
Cx26-17	2	22	34	95	2	93 (7.8 year)
Md (min-max)	2 (0–19)	19 (0–52)	19 (8–48)	27 (9–95)	2 (1–2)	99 (57–155)

Age at HI id., age when the child was identified with hearing impairment; Age at id. of etiology, age when the child's cause of deafness was identified;

* *Three children (CMV-10, Cx26-12, Cx26-16) had bimodal hearing (CI+HA); type of FCEI, family-centered intervention actions during the 1st year after 1st hearing aid fitting, with focus on individual parent guidance and with an auditory-verbal approach; 1 = Yes, on a regular basis; 2 = Yes, but not on a regular basis; 3 = No FCEI offered*.

#### Children With cCMV

All children (*n* = 10), six girls and four boys, had been screened at birth with OAE, and five children passed the first hearing screening without remarks. All children with cCMV were tested with an MR investigation before the CI intervention, and 100% (*n* = 10) had results that indicated slightly atypical patterns (white substance), mainly in the frontal regions of the brain (level 1 of three levels, where a higher level indicates more injuries) (Karltorp et al., 2014). The parents reported that there were no close family members with ADHD, ASD, or DLD. Some of the children with cCMV had been introduced and exposed to sign language or supported signs in daycare settings and in their home environment in early childhood. At the time of the follow-up study, however, only a few of them used signs themselves, and the majority of children went to mainstream preschools or schools. One child went to a special school for those with hearing impairment that had an adjusted listening environment, smaller class size, and spoken Swedish as the educational language. No child with a cCMV infection attended a deaf school. The nine children who attended mainstream schools had a certain degree of an adjusted listening environment in their mainstream classrooms. They were included in typical classes, with more pupils than in special schools, and there was a large variety in the type and level of support available for the individual child and their family.

#### Children With Cx26 Mutations

All children (*n* = 7), two girls and five boys, had been screened at birth with OAE. According to the parents, none of the families had close family members with ADHD, DLP, or ASD. All children communicated primarily with spoken language at home and in preschool/school. A few children knew and used sign-supported language or sign language. All but one had been going to mainstream daycare since they were toddlers, and they continuously went to mainstream preschools/schools, close to their homes, at the time of the study. One child with Cx26 attended a special school for hearing-impaired children. The rest of the group of children with Cx26 had a similar situation compared to children with cCMV who were mainstreamed (typical class sizes, some adjustment of the listening environment, a large variation in the type and level of individual child support in their preschool/school).

### Procedure

All participating families had visited the same Auditory Implant Center at Karolinska University Hospital since their child received their first CI. Families were scheduled for a duration of around 4 h at the follow-up occasion (see Table 2). The team at the Auditory Implant Center had previously assessed the child both pre- and post-implantation with a fixed test protocol and with the same test procedures. The participating children were randomly scheduled to meet a multidisciplinary team containing experienced clinicians/researchers: a medical doctor, speech-language pathologist, audiologist, social worker. In addition, a blinded psychologist who had no previous knowledge about the individual children and who did not know which group each participant belonged to (cCMV or Cx26) was included as a team member to perform the EF tests and behavior observations (Karltorp et al., 2014). Before the visit, parents and teachers had already filled out questionnaires that measured executive functions, pragmatics, and the mental health of the child. After the test occasion, the blinded psychologist, for validity reasons, observed the recorded video-based material from the test occasion. X-ray data (MR) and other child-related information regarding early clinical findings were retrieved from the individual children's medical records and were then reviewed in the data collection process by a medical doctor who was part of the multidisciplinary research team. The medical doctor met all families at the follow-up occasion for a short interview with the parents about their early FCEI services, family background (hereditary for ADHD and ASD etc.) and the child's medical health.

**Table 2:** Description of assessment tools used at different test occasions in the study.

**Assessment tools study**	**Preop**	**1 year with CI**	**3 years with CI**	**Follow-up**
**TESTS**				
Reynell-III (Language understanding)	X	X	X	
Expressive grammar scale	X	X	X	
SIR-2 (Speech intelligibility)	X	X	X	
BNT (expressive vocabulary)				X
Lexical-semantic error analysis (BNT)				X
FAS and Animal (word fluency ability)				X
Ravens (non-verbal cognitive ability)				X
TEA-Ch (attention level)				X
EBA-R (observational analysis scale)				X
SIPS; phonological working memory				X
SIPS; general working memory				X
Speech recognition (silence)				X
Speech recognition (noise)				X
**QUESTIONNAIRES**				
BRIEF (parents) (EF skills)				X
BRIEF (teachers) (EF skills)				X
CCC-2 (parents) (pragmatics)				X
SDQ (parent) (mental health)				X
SDQ (teachers) (mental health)				X

### Measures

#### Executive Functions—Tests, Questionnaire, and Qualitative Analyses of Behavior

##### Everyday attention level

Everyday attention level was assessed with the Test of Everyday Attention for Children (TEA-Ch) in children older than 6 years (Heaton et al., 2001; Manly et al., 2001). The TEA-Ch test has previously been translated to Swedish and used for other clinical groups, such as 7-years-old children with low birth weight (Starnberg et al., 2018), but there are so far no Swedish norms on the test. The TEA-Ch is a test that assesses everyday attention capacity and is presented both in an auditory or visual modality. The TEA-Ch consists of nine subtests; Sky Search, Score!, Creature Count, Sky Search DT, Map Mission, Walk- don't walk, Opposite Worlds, and Code transmission. These subtests assessed the participant's ability to sustain, select, and shift their attention (Manly et al., 2001). In the present study, the test procedure was conducted as suggested in the manual. The subtest Score Dual Task (to discriminate between two sound tracks only by listening) was excluded in the present study, for reliability reasons because it was too difficult to perform for participants with CI.

##### Phonological working memory

Phonological working memory (a non-word repetition task that is a relatively pure measure of the phonological loop capacity, Baddeley, 2012) and *General working memory* (the capacity to simultaneously store and process information, Wass, 2009) was assessed by using two subtests—Serial Recall on non-words and Sentence, Completion and recall, respectively—from the SIPS test battery (Wass, 2009) in children older than 5 years. In the Serial Recall on non-words subtest, children listened to standardized and recorded non-word material that was presented from loudspeakers and with gradually increasing numbers of non-words in a row. The children decided on the comfortable hearing level before the assessment. Then, participants were asked to repeat the non-word utterances as accurately as they could. The percentage of correctly reproduced consonants in the whole test was calculated. In the Sentence, Completion and recall subtest, the number of correctly recalled real words were counted. Examples of sentences were, “The sky is blue and the grass is…(green) (participant fills in)” and “You sit on a chair, and you sleep in a…(bed) (participant fills in).” Then, the test administer asks the participant, “Which words did you say?” These two cognitive tests have been used in children with TH and typical development and in clinical groups from around 6 years of age (Wass et al., 2008; Lyxell et al., 2009; Henricson et al., 2012).

##### Executive functioning in the home and a preschool/school environment

Executive functioning in the home and a preschool/school environment was rated in a questionnaire by parents and the child's primary teacher, respectively, who filled in the Behavior Rating Inventory of Executive Function (BRIEF) to evaluate possible behavioral problems concerning EF in everyday settings (at home and in preschool/school, respectively) (Gioia et al., 2000; Isquith et al., 2004). BRIEF functional scales were used to screen for possible behavioral problems in executive functioning in everyday life situations. The individual subscale results of BRIEF can be summarized in three different functional scales: Behavior Rating Inventory (BRI), Metacognition Index (MI), and the Global Executive Composite (GEC). Caregivers of all children filled out the questionnaire, as did the child's teacher (preschool or school). The BRIEF questionnaire has been translated to Swedish, but there is yet no validation of the test or Swedish norms available. We therefore compared the study results with the American norms. T-scores ≥65 were considered clinically significant, and any scores ≥70 were extremely high (Gioia et al., 2000; Isquith et al., 2004).

##### Emotional, behavioral, and attention rating (EBA-R)

Emotional, Behavioral, and Attention Rating (EBA-R), an in-house developed observational and qualitative analysis scale (Henricson and Löfkvist, Appendix 1), was used to evaluate the child's behavior during the test session with the psychologist (TEA-Ch). It was conducted by the blinded psychologist who also reviewed videotapes afterwards to confirm or adjust the initial observational rating results. Several categories were rated: Expression of positive emotions; Frustration level; Restlessness level; Focus level; Problem solving (structured ability, logical behavior); and Problem solving (unstructured ability, chaotic behavior) (see Appendix 1).

#### Pragmatic Skills

The second edition of the Swedish version of the parent report questionnaire Child Communication Checklist (CCC-2) was used to examine the children's pragmatic skills (Bishop, 2003). This assessment tool includes Swedish norms for children between 4–16 years (https://www.pearsonassessment.se/ccc-2). The checklist, which had 70 different statements, was filled in by parents and then analyzed afterwards with computerized scoring. The CCC-2 consists of 10 subscales; A–Speech; B–Syntax; C–Semantics; D–Coherence; E–Inappropriate initiations; F– Stereotypic language; G–Use of context; H–Non-verbal communication; I–Social relations; and J–Interests.

#### Mental Health

The SDQ is a 25-item screening questionnaire. Each item is rated 0 = not true, 1 = somewhat true, or 2 = certainly true (Goodman, 1997; Malmberg et al., 2003), in which 10 items reflect strengths, 14 reflect difficulties, and 1 is neutral but is scored as a difficulty item on the peer problems subscale (Goodman, 1997). A small number of negatively worded items are reverse scored. The items are grouped in five subscales containing five items each. The subscales are emotional symptoms, conduct problems, hyperactivity-inattention, peer problems, and prosocial behavior. Each subscale score ranges from 0 to 10. Higher scores on the prosocial behavior subscale reflect strengths, whereas higher scores on the other four subscales reflect difficulties. A total difficulty score is calculated by adding the sum of scores on the emotional, conduct, hyperactivity, and peer problems subscales, with a possible range of 0 to 40 (Goodman, 1997). The construction of cut-off values is based on normative SDQ scoring, as proposed by Goodman (1997). A total of 10% of a norm sample with the highest scores were classified as abnormal, the next 10% as borderline, and the remaining 80% as normal. These cut-offs varied between informant versions as well as across subscales and the total difficulties scale (Goodman, 1997, 2005). The psychometric properties of the Swedish parent-rated version of the SDQ have been evaluated by Smedje et al. (1999).

#### Speech Recognition

Sound field hearing thresholds were assessed by presenting frequency-modulated tones at octave frequencies from 0.125–6 kHz. The hearing tests were conducted using best-aided conditions (bilateral CI or in bimodal fashion; CI and HA) for speech in silence and in multi-source noise (Asp et al., 2012). The speech recognition in quiet was conducted with a 25-item list of monosyllabic words presented at 65 dB SPL level. The noisy conditions consisted of a presentation of stationary speech-shaped noise from ±45° to ±135° azimuth (uncorrelated signals), which resulted in a signal-to-noise ratio of 0 dB.

#### Screening of Non-verbal Cognitive Ability

All children were assessed with the Raven colored progressive matrices (Raven et al., 2003). This test evaluates an individual's ability to discover and interpret visual patterns and can be viewed as a screening tool for IQ. There are, so far, no Swedish norms on Ravens, and we therefore used the validated and standardized English norms for comparisons between participants and children with TH (Raven et al., 2003).

#### Language Abilities

Children were assessed by way of expressive vocabulary/picture naming by using a validated Swedish version of the Boston Naming Test (BNT) (Kaplan et al., 1983; Tallberg, 2005). The BNT has been normed for Swedish children aged 6–15 years (*N* = 152) (Brusewitz and Tallberg, 2010). The Boston Naming Test is an open-set test that consists of 60 pictures that the child is asked to name. In the current study, we did not allow phonological or semantic prompting. Synonyms and subordinated words were counted as correct words. A lexical-semantic error analysis was performed with the purpose of exploring more in-depth semantic knowledge of incorrect responses besides form scoring the number of correct responses on the BNT (Löfkvist et al., 2014). Word fluency tasks included Animal word fluency (semantically based) and FAS letter word fluency, a phonemically based task that not only measures word retrieval from the long-term memory but also targets EF indirectly, considering the individual's use of strategies in the process of retrieving words from their long-term memory. Both these two tests have been normed in Swedish children aged 6–15 years (*N* = 130) (Tallberg et al., 2011).

#### Early Language Abilities

The Reynell-III test evaluates expressive and receptive language abilities and was originally developed for children aged 0–7 years with TH (Edwards et al., 1997). A validated Swedish norm study of the *receptive test* part was conducted in a group of Swedish children with TH and typical development (Eriksson and Grundström, 2000). The results showed that children aged 2:6–3:5 years (*N* = 122) had comparable results with age-matched English children (Edwards et al., 1997). The Swedish norm data has a narrow age range. As the English and Swedish norm data showed similar results, it was therefore decided to use the English validated norms as comparisons with clinical data in the present study. Reynell-III was used to measure language understanding pre-op as well as after one year and three years post-op as part of the regular follow-up procedure for all children who have been implanted with CI at the Auditory Implant Center, Karolinska University Hospital, including the current study sample (Edwards et al., 1997).

Furthermore, experienced speech–language pathologists who were the same clinicians who performed the Reynell-III assessment pre-op, and after 1 and 3 years after the first CI, also rated the level of *expressive grammar* (level 1–8) and the child's level of *speech intelligibility* (see Appendix 2) (Allen et al., 2001; Löfkvist, 2014). The expressive grammar-rating scales (level 1: “no use of voice with intent” to level 8: “typical or correct expressive grammar and sentence level”) were developed within a Swedish context, primarily for use in children with HI, but may be used in other groups, including children with TH (see Appendix 2, Löfkvist, 2014).

The Speech Intelligibility Rating Scales (SIR-2) was specifically developed for use in children with HI and consists of a 5-level rating scale from “recognizable words in speech” to “connected speech is intelligible to all listeners” (Allen et al., 2001). The reliability of the SIR was originally tested and validated in 54 English children with CI, aged 1; 2–10 years. Experienced speech–language pathologists at the Auditory Implant Center at Karolinska University Hospital rated the SIR-2 before the first cochlear implantation and, thereafter, every 12 months until the child reached level 5. The SIR has been translated into Swedish and implemented at the Auditory Implant Center, but has not yet been validated in the Swedish context.

### Statistical Analyses

Potential group differences (cCMV infection vs. Cx26) were examined with Mann Whitney U-tests that included effect size indicators; *r* = Z/√N and a Chi-square test, and Spearman's correlations were used to examine the possible relationships between executive function, pragmatics, and mental health in the whole study sample (*N* = 17). As the sample size was small, and as it had a wide age range, only non-parametric statistical analyses were performed in the calculations. Individual data on BRIEF, phonological working memory, CCC-2, SDQ, and early language and speech intelligibility results after 3 years with the first CI are presented in Appendix 3.

## Results

We addressed three research questions in the current follow-up study that were related to possible similarities and differences in EF outcome, pragmatics, and mental health in a sample of deaf children with CI and with different etiological backgrounds. The groups (cCMV and Cx26) were initially matched based on age, hearing (CI), vocabulary (BNT; raw scores), and non-verbal cognitive ability (Ravens matrices). There were no statistically significant differences between groups (cCMV and Cx26) regarding the speech recognition outcome (**Table 4**), parent education level, early language abilities pre-op and after 1 year with the first CI (Table 3), or expressive grammar levels after 3 years with the first CI (*p* > 0.05) (Table 3). Nevertheless, there were two statistically significant group median differences for language understanding (Reynell-III) (Edwards et al., 1997) and speech intelligibility (SIR-2) (Allen et al., 2001) after 3 years, with better results for children with Cx26 mutations (Table 3). Individual test results on BRIEF, CCC-2, SDQ, and the two working-memory tasks are presented in Appendix 3.

**Table 3:** Early speech, language, and hearing outcome (pre-op, post-op after 1 and 3 years with 1st CI), and age at walking (months), on group level (cCMV infection and Cx26 mutations), including statistical values for group comparisons (Mann Whitney *U*-test, and calculated effect sizes).

**Abilities/tests (Md, min-max)**	**cCMV infection** ** (*n* = 10)**	**Cx26 mutations** ** (*n* = 7)**	***Z***	***p*-value**	***r***
**Language understanding**					
*(Reynell-III, raw scores)*					
Pre-op	0 (0–1), (*n* = 9)	0 (0–42)	−1.01	*p* = 0.31	0.25
1-year post-op	13 (3–43), (*n* = 9)	25 (17–53), (*n* = 6)	−1.89	*p* = 0.06	0.49
3- years post-op	47 (37–52), (*n* = 9)	51 (51–54), (*n* = 5)	−2.09	*p* = 0.04	0.56
**Speech intelligibility rating**					
*(SIR-2, clinical judgement)*					
Pre-op	1 (1–5)	1 (1–4)	−0.69	*p* = 0.49	0.17
1-year post-op	2 (2–3), (*n* = 9)	3 (2–4), (*n* = 6)	−1.86	*p* = 0.06	0.45
3-years post-op	4 (2–5), (*n* = 9)	5 (4–5)	−2.08	*p* = 0.04	0.50
**Expressive grammar level**					
*(EGL, clinical judgement)*					
Pre-op	2 (1–8)	2 (1–7)	−0.41	*p* = 0.68	0.10
1-year post-op	6 (4–7), (*n* = 9)	6 (5–8), (*n* = 6)	−1.05	*p* = 0.30	0.27
3-year post-op	7 (7–8), (*n* = 9)	8 (7–8)	−1.39	*p* = 0.17	0.34
**Age at walking (months)**	18 (12–23), (*n* = 9)	12 (11–13)	−3.05	*p* = 0.002	0.76

*Language understanding; Reynell-III (Edwards et al., 1997); Speech Intelligibility Rating (SIR-2) (Allen et al., 2001), rating scale 1–5; Expressive Grammar Level (EGL), rating scale 1–8 (Löfkvist, 2014); Pure-Tone Average (PTA) performed with best aided situation; Age at walking, information from parents*.

**Question 1:** *Do children with CI have worse EF results in relation to norm data, regardless of cause of deafness, and do children with a cCMV infection have even poorer executive functioning compared to children with genetic non-syndromic deafness (Cx26)?*

### Executive Functioning on Tests (Working Memory, Attention)

#### Working Memory

There was one statistically significant difference between children with cCMV and children with Cx26 on the phonological working memory test (*Z* = −2.30, *p* = 0.02, *r* = 0.56), with worse results for children with cCMV, while there were no statistically significant differences between groups on general working memory (*Z* = −0.95, *p* = 0.34, *r* = 0.23).

#### Attention Level

Attention level was assessed with the TEA-Ch test in all children older than 6 years. Although, there were only six children with cCMV and four children with Cx26, one statistically significant difference was found on one subscale; “walk don't walk” targets impulse control under time pressure (*Z* = −2.0, *p* = 0.04, *r* = 0.63). Due to missing data on some subtests for a few individuals and in combination of the small numbers in the sample, it was not possible to further evaluate whether the results were comparable or worse than for peers with TH in the same ages (norm data).

#### Emotional, Behavioral, and Attention Rating (EBA-R)

There were no statistically significant group differences on any of the scales: Expression of positive emotions; Frustration level (*Z* = −1.61, *p* = 0.11, *r* = 0.39); Restlessness level (*Z* = −1.49, *p* = 0.14, *r* = 0.36); Focus level (*Z* = −1.30, *p* = 0.20, *r* = 0.32); Problem solving (structured ability, logical behavior) (*Z* = −1.93, *p* = 0.05, *r* = 0.49); or Problem solving (unstructured ability, chaotic behavior) (*Z* = −1.69, *p* = 0.09, *r* = 0.41).

### Executive Functions in Everyday Settings (Home and Preschool/School)

**Figure 1:**
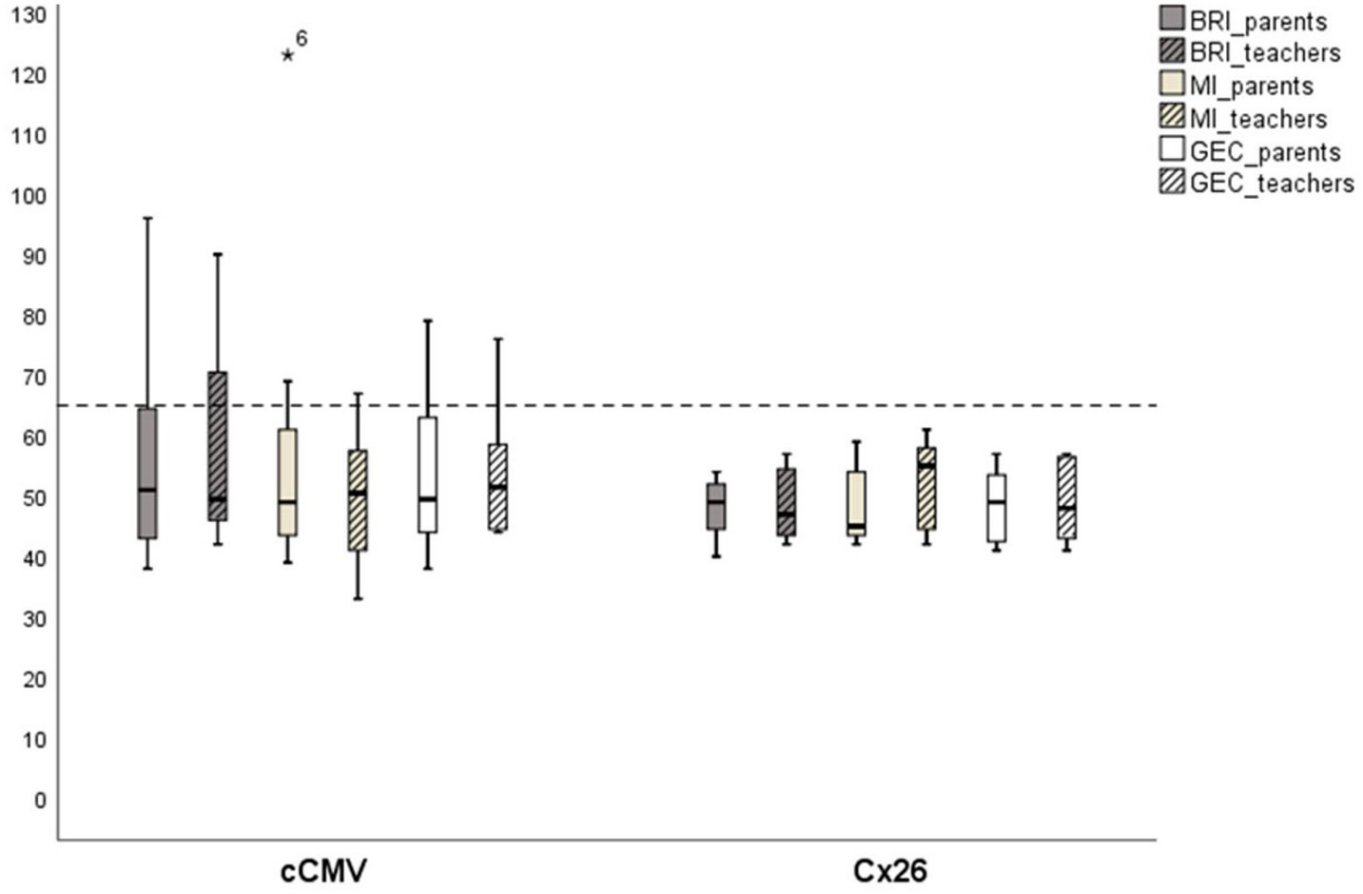
*Executive functions in home and pre-school/school environment (BRIEF-results)*. Children deafened due to congenital cytomegalovirus (cCMV) infection (*n* = 8) and connexin 26 (Cx26) (*n* = 7), showing *t-*score results on group level; Behavior Rating Inventory (BRI), Metacognition Index (MI), and Global Executive Composite (GEC), rated by parents and teachers. Scores over 65 is considered to be clinically atypical. *Participant 6.

**Figure 2:**
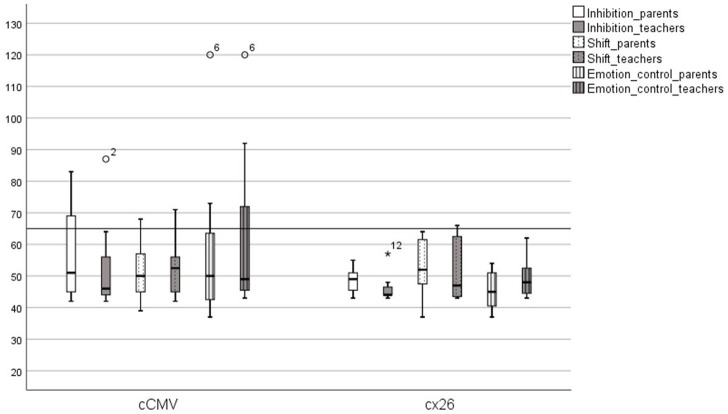
*Behavioral Regulation Scales (BRIEF-results)*. Children with congenital cytomegalovirus (cCMV) infection and children with connexin 26 (Cx26), showing *t*-score results on the following scales; inhibition, shift, and emotion control, rated by parents and teachers. *Participant 12, °participants 2 and 6.

The group median results of the BRIEF rating indicated slightly worse results than expected in relation to norm data for children with TH, but there was a large variation within the cCMV group. The majority of children with cCMV were within limits of typical levels compared to American norm data. We found no statistically significant group differences (cCMV and Cx26) (*p* > 0.05). Nevertheless, there were three individuals with cCMV who had poorer EF results than controls and in relation to norm data, which should be examined further in more in-depth investigations by a clinical psychologist (see Figures 1, 2).

**Figure 3:**
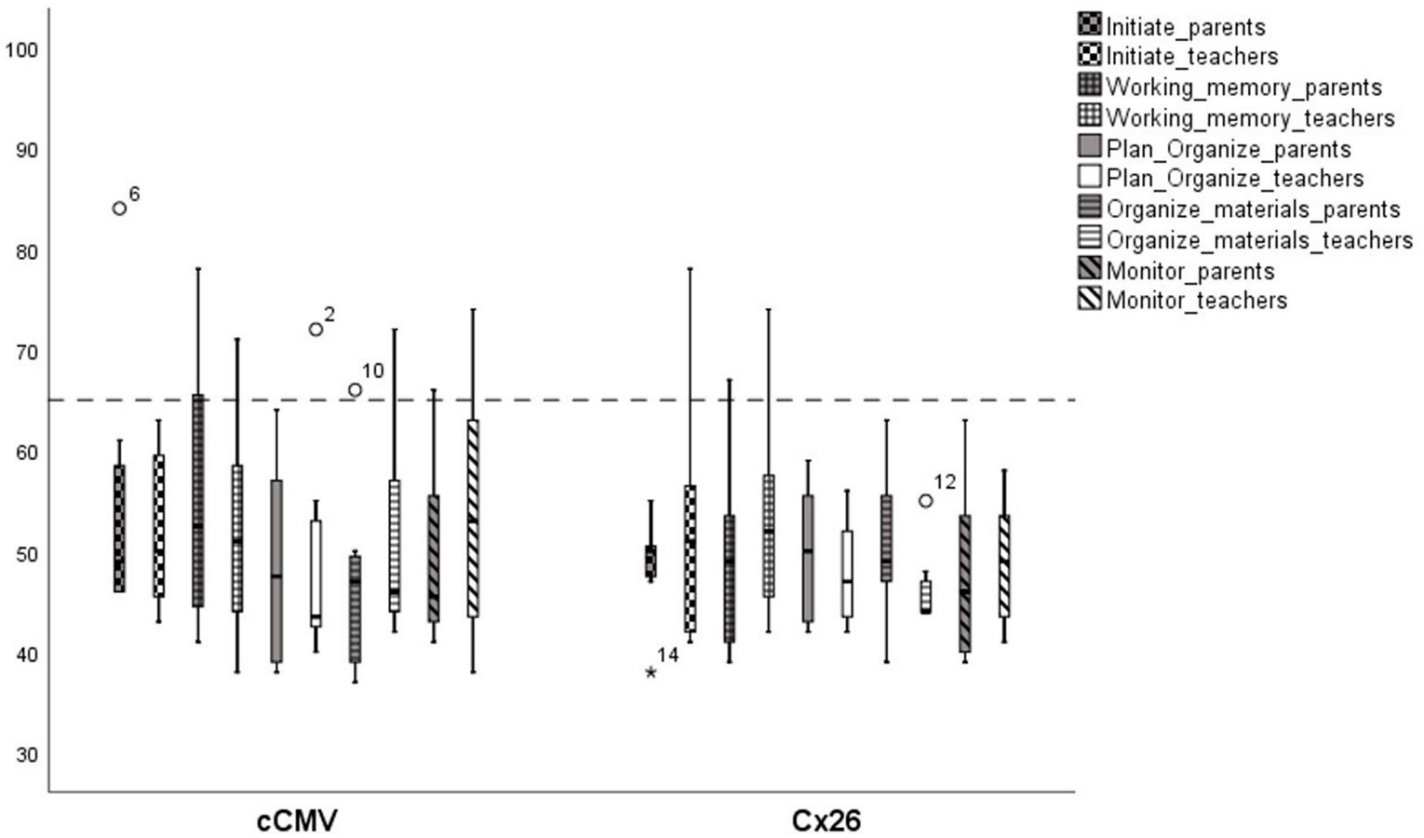
*Metacognition Scales (BRIEF-results)*. Children with congenital cytomegalovirus (poX) infection (*n* = 8) and children with connexin 26 Cx26 (*n* = 7), showing *t*-score results on the following scales; initiate, working, memory, plan/organize, organize, and monitor, rated by parents and teachers. Scores over 65 is considered as clinically atypical. *Participant 14, °participants 2, 6, 10, and 12.

Although there was some variation in outcome between individuals within the Cx26-group, there was no child with genetic deafness who reached a t-score over 65 on either the BRI, MI, or GEC, indicating that children with Cx26 were within typical levels for children with TH in the same ages (norms). This suggests that children with Cx26 deafness did not have specific EF problems at home or in preschool/school. One child with Cx26 had results that scored higher than average on working memory and shifting (two subscales in BRIEF), which means that this child could have slightly worse results than expected, but not clinically atypical (see Figures 1–3).

To summarize, our first hypothesis that children with CI in both groups (cCMV and Cx26) had worse EF outcomes than children with TH was only partly confirmed by these pilot results. Children with cCMV had statistically significant worse phonological memory abilities than children with Cx26. Due to the small sample size and missing data from the TEA-Ch test we could not conclude that children with cCMV had substantially poorer attention and impulse control than children with Cx26 mutations. Three individuals with cCMV had BRIEF results that indicated they should be referred to a clinical psychologist for a more thorough investigation of their EF, while there were none in the control group with similar indications.

**Question 2:** *Do children with a cCMV infection who use CI have worse pragmatic skills and mental health status than well-matched children with Cx26 in comparison to norm data?*

#### Pragmatic Skills

**Figure 4:**
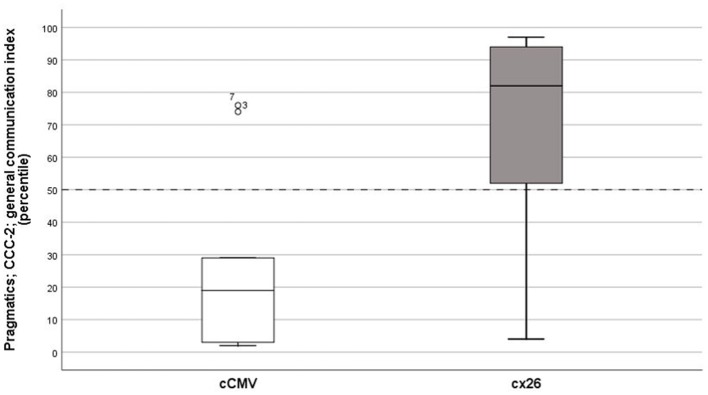
*Pragmatic skills*. General communication index, median percentile score on the Children's Communication Checklist (CCC), second edition, on group level congenital cytomegalovirus (cCMV) infection; *n* = 9 and connexin 26 (Cx26); *n* = 7.

**Figure 5:**
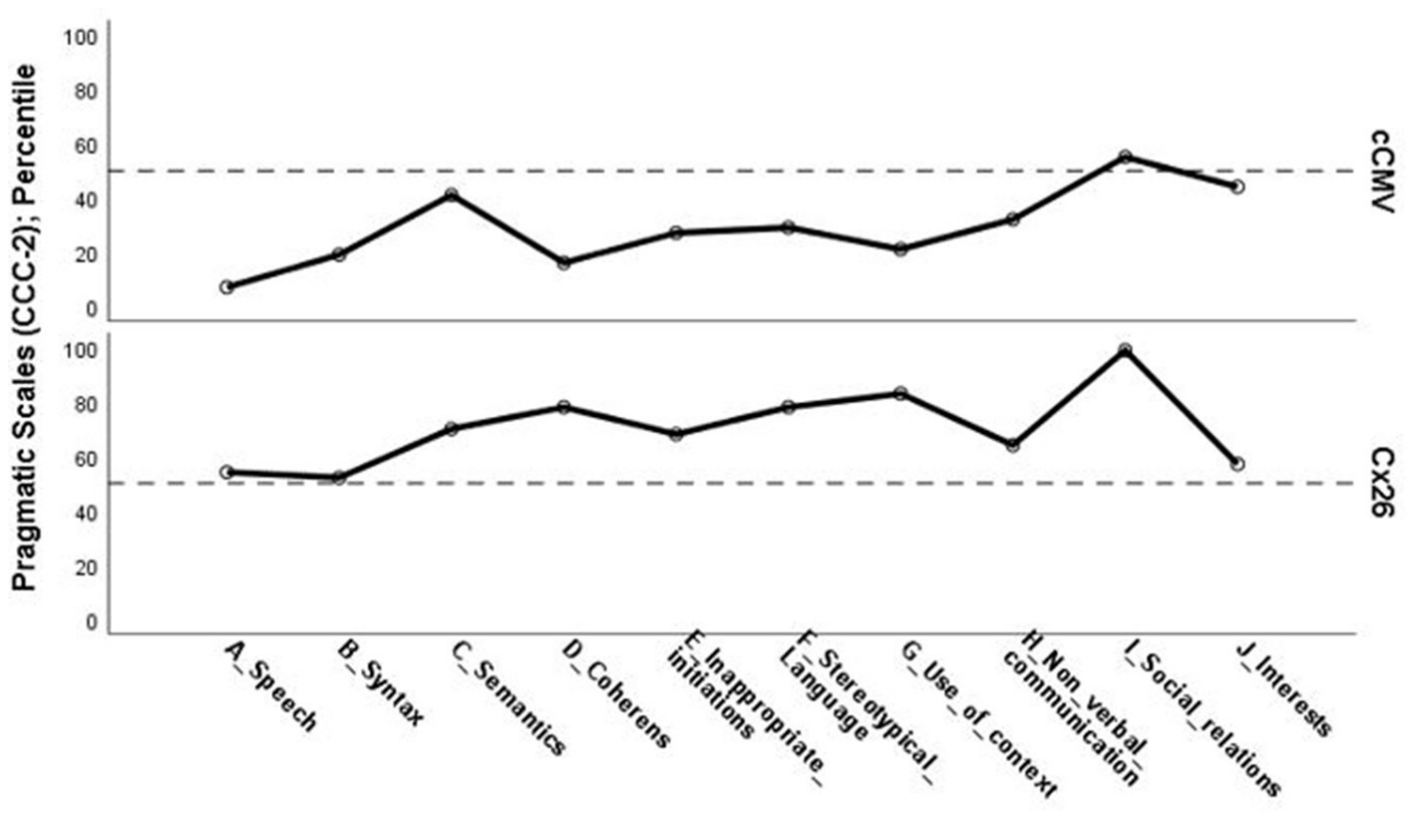
*Pragmatic skills*. Percentile results (Md) on individual scales on the Children's Communication Checklist (CCC), second edition, on group level; congenital cytomegalovirus (cCMV) infection: *n* = 9, and connexin 26 (Cx26): *n* = 7.

Results on the parent questionnaire CCC-2, measuring the child's pragmatic skills, showed significant differences between groups (cCMV infection and Cx26) on the IGK/total raw score (*Z* = −2.28, *p* = 0.02, *r* = 0.57), and on two subscales; Initiatives (*Z* = −2.40, *p* = 0.02, *r* = 0.60), and Use of context (*Z* = −2.87, *p* = 0.002, *r* = 0.72) (Figures 4, 5).

#### Mental Ill Health

All children in the sample, with a few exceptions, had typical results on mental health (SDQ) compared to norm data (Table 4). There were only three statistical differences between groups (cCMV and Cx26) on the SDQ results for individual subscales for results reported by fathers, which was related to more conduct problems and peer problems in the group of children with cCMV (see Table 5).

**Table 4:** Language, non-verbal cognition and hearing outcome measures (median, range, and statistical values), compared on group level (cCMV vs. Cx26), including the effect sizes.

	**cCMV (*n* = 10)**	**Cx26 (*n* = 6)**	***Z***	***p*-value**	***r***
**Vocabulary (BNT)**					
Raw scores	31 (0–46)∞	34 (23–53)	−0.82	*p* = 0.43	0.05
Stanine	4 (1–9)∞	3 (1–9)	−0.48	*p* = 0.63	0.02
**Lexical-Semantic error analysis (BNT)**					
Semantic relevant errors	11 (6–24)∞	11 (6–22)	−0.42	*p* = 0.68	0.01
Semantic irrelevant errors	11 (3–22)∞	10 (0–21)	−0.53	*p* = 0.60	0.07
No responds	0 (0–13)∞	0 (0–8)	−0.14	*p* = 0.89	<0.01
**Phonemic word fluency (FAS letter WF)**					
Total numbers	18 (0–34)	13 (1–37)^*^	−0.20	*p* = 0.84	<0.01
**Semantic word fluency (Animal WF)**					
Total numbers	13 (2–19)	10 (7–17)	−0.33	*p* = 0.74	<0.01
**Non-verbal cognitive ability**					
Raw scores	25 (14–34)	32 (14–35)	−1.36	*p* = 0.17	0.12
**Speech recognition**					
Quiet (%)	84 (64–100)§	68 (48–100)	−0.83	*p* = 0.41	0.05
Noise (%)	50 (32–68) ×	56 (32–68)¤	−0.52	*p* = 0.60	0.02

*Missing data in the Cx26-group: one child (Cx16) only participated with parent questionnaires*,

* *(n = 5)*,

*^¤^(n = 4). Missing data in the cCMV-group; ^§^(n = 9); ^×^(n = 8); ^∞^(n = 9); one child (CMV2) did not want to participate in the BNT for unknown reason, and therefore there are also missing data on the lexical-semantic error analysis for one individual*.

**Table 5:** Mental health.

		**cCMV** **(*n* = 9)**	**Cx26** **(*n* = 7)**	***Z***	***p*-value**	***r***
**SDQ—total score**	F	6 (2–12)	3 (0–7)	−1.75	*p* = 0.08	0.44
	M	6 (0–12)	3 (0–9)	−1.01	*p* = 0.31	0.25
	T	4 (0–16)	3 (0–10)	0.00	*p* = 1.00	–
**A. Emotional symptoms**	F	0 (0–2)	1 (0–2)	−1.01	*p* = 0.31	0.25
	M	0 (0–3)	1 (0–4)	−1.74	*p* = 0.08	0.44
	T	4 (0–16)	0 (0–3)	−0.86	*p* = 0.39	0.22
**B. Conduct problems**	F	2 (0–3)	0 (0–1)	−2.23	*p* = 0.03	0.56
	M	1 (0–3)	0 (0–3)	−0.82	*p* = 0.41	0.21
	T	0 (0–3)	0 (0–2)	−0.15	*p* = 0.88	0.04
**C. Hyperactivity**	F	3 (1–6)	0 (0–1)	−1.51	*p* = 0.13	0.38
	M	3 (0–8)	0 (0–5)	−1.39	*p* = 0.16	0.35
	T	2 (0–10)	2 (0–7)	−0.89	*p* = 0.39	0.22
**D. Peer problems**	F	1 (0–3)	0 (0–1)	−2.12	*p* = 0.03	0.53
	M	0 (0–5)	0 (0–1)	−0.42	*p* = 0.68	0.11
	T	0 (0–2)	0 (0–1)	−0.43	*p* = 0.67	0.11
**Prosocial behavior**	F	8 (6–10)	10 (7–10)	−1.94	*p* = 0.05	0.49
	M	9 (7–10)	10 (8–10)	−0.88	*p* = 0.38	0.22
	T	7 (5–10)	9 (2–10)	−0.07	*p* = 0.95	0.02

*Total score on SDQ and scores on subscales (Md, min-max) on group level (cCMV and Cx26), including Md group comparisons (Mann Whitney) and effect size calculations. F, fathers; M, mothers; T, teachers. Missing data in cCMV-group: reports from one mother (n = 8), two fathers (n = 7) and three teachers (n = 6). Missing data in Cx26-group: report from one father (n = 6). Higher scores on total score and subscales A–D indicate more difficulties, while higher scores on the prosocial behavior subscale reflect strengths*.

The second hypothesis we had before the study was that children with cCMV would have worse pragmatic skills than hearing-matched controls due to (presumed) worse executive functioning. The results showed statistically significant differences between groups (cCMV and Cx26), both on total raw score and on subscales that are related to conversational skills (initiatives and use of context); both are important for social cognition and could be related to *attention skills* and *flexibility* (EF). We hypothesized that worse pragmatic skills in children with cCMV could be explained by not only their auditory deprivation and HI but also by other consequences related to atypical MR findings and a congenital virus infection, which is known to be associated with other deficits (Karltorp et al., 2014). The results indicated that the statistically significant group differences and effect sizes on CCC-2 were not only explained by the HI only but by other reasons too. However, the sample size was small, which made it difficult to generalize the findings on population level.

**Question 3:** *Is there a relationship between EF, pragmatic skills, mental health, and early language abilities in children with CI, regardless of cause of deafness?*

#### Correlation Analyses

There were some correlations among EF, pragmatic skills, mental health, and level of language understanding and speech intelligibility rating after 3 years with the first CI, and these are presented in Table 6. These results represent the whole sample (cCMV and Cx26). The results showed statistically significant correlations both between the higher level of pragmatic skills and early language abilities as well as for pragmatic skills, mental health levels rated by parents, and some weaker correlations with phonological working memory (Table 6).

**Table 6:** Correlation coefficients for EF skills, pragmatics and mental health rated by mothers and fathers, and early language abilities after 3 years with 1st CI; language understanding (Reynell-III) and speech intelligibility (SIR-2), for children with cCMV and Cx26.

	**1**	**2**	**3**	**4**	**5**	**6**	**7**
1. Phonological WM	–	−0.56^*^	0.61^*^	0.55	0.39	−0.48	−0.65^*^
2. EF skills, GEC (BRIEF)		–	−0.58^*^	−0.42	−0.34	−0.70^**^	0.38
3. Pragmatics (CCC-2)			–	0.74^**^	0.63^*^	−0.73^**^	−0.63^*^
4. Language understanding (Reynell)				–	0.65^*^	−0.48	−0.48
5. Speech intelligibility (SIR-2)					–	-32	−0.43
6. SDQ, total score, M						–	0.65^*^
7. SDQ, total score, F							–

*EF skills, GEC, executive function skills, Global Executive Composite in BRIEF. M, mothers; F, fathers. Missing data from individual tests are reported in Tables 3–5*.

* *p ≤ 0.05*;

** *p ≤ 0.01*.

One initial hypothesis was that there would be a relationship between EF, pragmatic skills, and mental health in all children with CI, regardless of their cause of deafness, and that better speech and language understanding in early childhood would be related to better outcomes in pragmatics, EF, and social behavior in later childhood (Goberis et al., 2012; Hintermair, 2013). Apparently, children with cCMV showed more of that expected interaction pattern, but, because of the small sample size, the statistical correlation results had to be interpreted with caution, and the relation did not showing a casual effect.

## Discussion

In this follow-up study of children with cCMV compared to well-matched controls, we started with three research questions that arose after previous findings indicated that children with cCMV might have specific EF difficulties, which could affect their social or pragmatic development/behavior (Karltorp et al., 2014). As a group, most participants had age-adequate EF results compared to American norms for TH children concerning EF in everyday settings, rated by parents and teachers, which was somewhat surprising considering previous findings in the literature (Figueras et al., 2008; Kronenberger et al., 2014; Korndewal et al., 2017). When looking more closely at the subgroup patterns (cCMV vs. Cx26), and in relation to individual results, there were three children with cCMV who did not perform like typically developed children with TH and who should therefore be referred to a clinical psychologist to conduct more in-depth investigations of their EF.

On a group level, children with cCMV did have statistically significant worse phonological working memory than matched controls, but there was no group difference on general working memory. This indicates group-specific differences in how linguistic information is processed. Children with cCMV appeared to find it especially more difficult to process phonologically based information without semantic clues than children with Cx26. The children in the whole sample showed variation in the vocabulary outcome, but there were no statistically significant subgroup differences regarding the vocabulary size (total score on BNT) or the lexical-semantic error response analysis. Children with cCMV performed well on the FAS letter-fluency task, which means that children in the sample (on a group level) had sufficient and effective strategies to learn words and retrieve lexical-semantic information from their long-term memory despite the fact that they also had a worse ability to process non-words (Löfkvist et al., 2012; Löfkvist, 2014).

Some individuals with a cCMV infection did not complete all the tests, due either to fatigue or for unknown reasons, while children with Cx26 did not complain about fatigue in the same way, indicating worse *attention abilities* in children with a cCMV infection. Still, the EBR observation performed by the blinded psychologist did not show statistically significant group differences in performance during the test situation while performing cognitive tasks. One limitation was that the EBR observation was only performed in one test situation. It would have been useful to perform the same kind of observation also during the language and hearing assessment to further explore possible group differences related to the fatigue and attention level of participants during other assessments at the same test occasion. The attention measures in the TEA-Ch test were especially difficult to interpret, mainly due to few completed test results. The discovery of fatigue and attention difficulties in participants who did not complete the TEA-Ch-test suggests that a more sensitive measure of attention may be more informative to use in future studies. The result, however, also indicated that the TEA-Ch-test was challenging for all participants because it was assessing attention skills (Figueras et al., 2008; Beer et al., 2014; Kronenberger et al., 2014).

Children with CI, regardless of their cause of deafness, had more or less typical mental health results that were comparable to norm data of age-matched children with TH, which is a positive result. Only two aspects related to mental health differed in the two subgroups. Fathers reported conduct problems and poor peer functioning in children with cCMV compared to the hearing-matched controls with Cx26, which could be related to worse EF (Lyxell et al., 2009) and/or poorer pragmatic skills (Goberis et al., 2012).

There were statistically significant differences between groups on the total score of the pragmatic skills questionnaire (CCC-2) as well as for the subscales *initiatives* and *use of context* (in dialogues). Children with cCMV appeared to have worse ability to make use of the context in social interactions, and, according to parent reports, they used more initiatives that were irrelevant in verbal interactions despite having a similar language level and non-verbal cognitive ability as children with Cx26. The children with cCMV could be at risk of having more affected pragmatic skills than controls due to a later HI diagnosis age, resulting from their progressive hearing loss, and deviant pragmatic skills that were more related to their congenital CMV infection and atypical brain patterns (Karltorp et al., 2014).

Most et al. (2010) investigated pragmatic skills in a sample of 24 children with HI aged 6; 3–9; 4 years who had CI (*n* = 11) or used hearing aids (*n* = 13) and with 13 controls with TH. The pragmatic skills were similar for all participants with HI regardless or type of hearing technology. On a group level, children with HI had statistically significant poorer outcomes than children with TH. The authors concluded that their less effective pragmatic skills could be explained by impaired auditory perception of spoken language, less flexible use of language in combination with deficits in theory of mind, less exposure to different pragmatic situations, and poor use of repairing strategies. An additional explanation for their delayed pragmatic skills was their late diagnosis of HI (1; 8 years), which was influenced negatively by prolonging the length of auditory deprivation, especially for the deaf children who had a mean age of 2; 6 years when they received their first CI. Cause of deafness was not investigated in the study (Most et al., 2010). The study findings by Most et al. (2010) showed that children with late identification and management of HI had a more delayed acquisition of pragmatic competence, which could lead to consequences, not only in social interaction with friends, but also in learning situations.

The fathers in the current study reported statistically significant less-well conduct levels and peer problems in the cCMV infection group compared to controls. These two functions are interrelated and associated with social behavior. Poor behavior could lead to affected peer relations. Conduct level might also be related to pragmatics (Goberis et al., 2012), EF abilities like attention and phonological working memory (Lyxell et al., 2009), as well as social behavior (Hintermair, 2013). We found some correlations in the whole sample (cCMV and Cx26) between early language skills after 3 years with the first CI and later outcomes at the follow-up study, not only with pragmatic skills but also with phonological working memory and mental health. Better early language skills were associated with better pragmatic skills and phonological working memory at later ages. Better pragmatic skills were also related to better mental health. These findings should be investigated further in larger groups (cCMV and Cx26) to find out if there are more specific subgroup differences and if this has any relation to the children's own perceived mental health. In the current study, only parents and teachers responded on behalf of their child. An analysis of the child's own perceived mental health could give another result with worse self-perceived mental health in comparison to the view of the child's parents, which has been reported in previous studies of children with CI (Anmyr et al., 2012).

The participating children in the two groups were initially matched based on age, hearing-level, vocabulary knowledge, non-verbal cognitive ability, home language situation (at least one parent who speak Swedish), and no other known additional diagnoses besides the deafness. Furthermore, at the time of the follow-up study we found no differences between groups based on socio-economic status (parental education level). Nonetheless, there were some significant statistical group differences between children that were related to their early childhood. Children with cCMV on average started to walk later than children with Cx26, which is suggestive of a balance problem that has been reported on before (Karltorp et al., 2014). They also had statistically significant worse language understanding and speech intelligibility after 3 years with their first CI compared to the results of controls with Cx26 (Ramirez Inscoe and Nikolopoulos, 2004; Yoshida et al., 2009), while there were no group differences after 1 year with their first CI. Apparently, children with a cCMV infection developed their spoken language at a slower pace than children with Cx26 despite there being a possible better hearing situation as infants in some cases of cCMV infection due to the late onset of HI, which is a result that has been reported on before (Ramirez Inscoe and Nikolopoulos, 2004; Yoshida et al., 2009; Karltorp et al., 2014). In a follow-up study by Yoshida et al. (2017), in 16 children with a cCMV infection and with a mean follow-up time of 7.8 years after the first CI, the authors found that some children who had initial delayed language had caught up in speech and language understanding. Yet, there were some children who instead showed increased difficulties related to the incidence of additional diagnose(s) and more brain abnormalities in infancy (Yoshida et al., 2017), which is similar to the results of the current study, with a large variation of outcome, especially in the cCMV group.

Another difference between groups (cCMV and Cx26) in the current study was their early daycare environment. All but one child with Cx26 went to mainstream daycare from the start and continued to be mainstreamed onwards, while some children with cCMV infection had initially attended special units for deaf and hearing-impaired children, with more exposure to sign language and total communication, before they changed to mainstream preschools/schools. All participants with cCMV had parents who were TH; all children with cCMV therefore had access to spoken language throughout their early childhood in their home environment. We therefore have no reason to believe that the initial different daycare settings would explain later group differences in EF abilities or language outcome, including their pragmatic skills. The worse spoken language understanding level after 3 years with CI in children with cCMV infection could potentially be related to limited exposure of spoken language in daycare, but is more likely explained by previous findings that there is a slower pace in speech and language development in children with cCMV compared to other subgroups of children with CI (Ramirez Inscoe and Nikolopoulos, 2004; Yoshida et al., 2009).

### Study Limitations and Future Studies

Although the present study was limited in the number of individuals, the pilot study contributed with new knowledge about executive functioning, pragmatic skills, and mental health in deaf children with cCMV who use CI as well as for matched controls with Cx26 mutations. Future studies should look more closely into individual results in children with a cCMV infection. It would be beneficial to conduct a study with a longitudinal study design to further examine the developmental aspects of executive functions and pragmatic skills and include theory-of mind as an aspect in relation to the children's mental health, including their own self-perceived opinion and perspectives. Comparative cross-sectional studies should include more participants with cCMV and controls with TH who are matched based on age and socio-economic status and preferably also including a control group of typically hearing children with ADHD.

To conclude, children with a cCMV infection who used CI, and who did not have previous known diagnoses like ADHD, DLD, or ASD, had worse pragmatic skills and phonological working memory compared to well-matched controls with Cx26 and CI. Both groups with CI had typical mental health according to parent and teacher reports; some fathers' reports, however, showed more conduct problems and poor peer functioning in the group of children with cCMV infection. Parents and teachers did not report severe EF difficulties in everyday settings on group level. Better early language skills after 3 years of CI use was correlated to better pragmatic skills and mental health at later ages. The results indicate that it is important to identify children with cCMV as early as possible and support them and their families with preventive language stimulation actions, including specific training of social and pragmatic skills. Besides listening and language abilities, social cognition and EF should be assessed on a regular basis. This might limit the risk that subgroups like children with cCMV are left behind in social interaction and learning situations.

## Data Availability Statement

The datasets generated for this study are available on request to the corresponding author.

## Ethics Statement

This study was carried out in accordance with the recommendations of the Regional Ethical Review Board in Stockholm, Sweden. All participants were first provided with written information about the study. Then a written informed consent was obtained from the parents of all participants, in accordance with the Declaration of Helsinki. The protocol was approved by the Regional Ethical Review Board in Stockholm, Sweden; DN 2012:/2.

## Author Contributions

All authors contributed with planning of the study, conducted the data collection, and performed data analysis/interpretation as well as discussed results and commented on the manuscript at all stages. UL conducted most of the statistical analysis as well as prepared the draft.

### Conflict of Interest

The authors declare that the research was conducted in the absence of any commercial or financial relationships that could be construed as a potential conflict of interest. The handling editor declared a shared affiliation, though no other collaboration, with one of the authors CH at the time of the review.
