# Supplementary material for: Executive Functions, Pragmatic Skills, and Mental Health in Children With Congenital Cytomegalovirus (CMV) Infection With Cochlear Implants: A Pilot Study
Source: Front Psychol. 2020 Jan 10;10:2808. doi: 10.3389/fpsyg.2019.02808 (PMC6965306; doi:10.3389/fpsyg.2019.02808)
Supplement: Supplementary file 1 [file Data_Sheet_1.docx]

**Appendix 1. Emotional, Behavioral & Attention Rating (EBA-R) (Observational Qualitative Analysis) (Henricson & Löfkvist)**

The rating scale was developed by a psychologist (test administrator of the TEA-CH) who observed and rated emotional, behavioral and attention abilities both during the testing session and from videotapes afterwards to verify results.

**Description of EBA-R Categories Rating Scale Level**

**Expression of positive emotions: 1-5**

Estimation of the extent to which the child presents with positive expressions during the test occasion and how well they harbor positive emotions.

1. The child is neutral, exhibits no special expressions and gives very little information about his/her satisfaction level.

2. The child is mostly neutral. Smiles when given positive feedback about participation, or spontaneously expresses satisfaction when something interesting occurs during the testing session.

3. The child is positive and seems to be satisfied. Often smiles, and is strengthened by positive reinforcement from the test administrator, and expresses some own interest.

4. The child is laughing without any obvious reason, becomes eager when given positive feedback, and throws himself/herself over new tasks.

5. The child often laughs, constantly commits himself/herself (relies on the fact that recent experiences of mastery will lead to improvement next time) and becomes even more positive with positive feedback.

**Frustration level: 1-5**

Estimation of the extent to which the child shows signs of frustration

during the test and how well they can accommodate it.

1. The child is neutral, exhibits no particular expressions and gives very little information about a potential frustration level.

2. The child is mostly neutral. Sighs when it seems to be hard to complete a test part or to participate in tasks. Wrinkles the forehead at occasions.

3.The child is mildly frustrated. Has some sour expressions, sighs, and hesitates before tasks. Needs some encouragement from the test administrator to become motivated.

4. The child is rather frustrated during testing. Whines intermittently, indicating that the tasks are boring and/or difficult. Sighs and puffs. Often needs encouragement from the test administrator to become motivated.

5. The child is very frustrated. Has a lot of sour expressions and whines, sighs, puffs and moans because of the tasks. Needs a lot of encouragement to become motivated. Seems to give up sometimes during testing and/or ignores the instructions and rules.

**Restlessness level**:  **1-7**

Estimate the child's ability to sit still during the test administration while not showing signs of restlessness.

1. Sits down calmly. Only changes their seating position occasionally, usually between tasks.

2. Almost sits down calmly. Change include collapsing on the chair and stretching out from time to time. Changes seating position somewhat more often, and with somewhat worse timing (during the tasks).

3. Intermittent restlessness in the body. Swivels slightly, and changes seating position during testing. Moves their hands from time to time, e.g. scratches himself/herself.

4. Mildly restless. Swivels slightly on the chair. Often changes seating position during the test-situation. Has active hands that pick, scratch and move.

5. Restless but can still control it. Swivels a lot, kicks with the legs, changes seating position very often, stretches. Their hands are moving around all the time.

6. Very restless during tasks as well as in between tasks during the testing session. Needs to take breaks.

7. Can hardly sit still at all. The child is constantly moving around in the room or is almost falling down from the chair because of restlessness. Playing with objects on the table that are not involved in the task. The child has to be stopped from grabbing the test material.

|  |
| --- |

**Focus level: 1-5**

Estimate the child's ability to focus on the tasks in the test and maintain focus during the test administration.

1. The child is totally focused on the tasks. Seems to pay close attention to instructions and asks relevant questions if anything is unclear. Keeps this focus throughout the test session.

2. The child is focused on the tasks and listens carefully to the test administer. Some signs of tiredness during testing, e.g. makes minor mistakes during tasks which last longer than others. Shows signs of tiredness at the end of the test session (yawns, and requests clarification more often).

3. The child seems to be focused on the tasks and during longer activities, but sometimes loses focus and forgets to answer, or needs the assessor to repeat the instructions again. Becomes tired if the tasks are longer and is apparently tired after the testing.

4. The child has difficulties to focus for longer periods during the testing session. Asks about irrelevant things or wants to know how much time is left of the test/tasks. Needs repeated instructions. Becomes obviously tired by the longer tasks and therefore misses some raw scores in the test. Very tired after the testing session.

5. The child cannot focus more than a few moments at a time (30-40 sec). Forgets the instructions, despite repetition. Many questions about how much time there is left, and when they are finished with the test. Rubs the eyes and face, yawns and often asks “what?”. Randomly gives the right response on test questions, among many wrong ones. The child is unable to complete all the test parts or cannot complete all the tasks.

|  |  |  |  |  |  |  |
| --- | --- | --- | --- | --- | --- | --- |
|  | | | | | | |

**Problem Solving level; structured ability, logical behavior:** **1-5**

Estimating the child's ability to handle the data in a structured and logical manner, e.g. to use strategies and adapt them based on new conditions.

1. The child understands the instructions and has a plan for the tasks. Deals with the tasks with a certain “calm” and obvious strategy (e.g. seeks in a Z-pattern over half the picture during the Spaceshipshunt, (TEACH-test)). If the child discovers that the strategy is not working well (e.g. that the child has a hard time to follow the tempo during certain tasks) he/she tries to revise that strategy.

2. The child has a strategic plan based on the given instructions. Is calm in the beginning but has some difficulties to adjust to a new strategy if the previous strategy does not work.

3. The child seems to try, and use strategies based on the instructions, but has difficulties to judge if the strategy is working well and/or if changes in the strategies are necessary.

4. The child creates a strategy during the performance of the task. Seems to have difficulties judging if the strategy is successful or not. Seems to change strategies often.

5. The child lacks any strategy and does not create any new strategies during the test. Handles each question during different tasks differently.

**Problem Solving level; unstructured ability, chaotic behavior:** **1-5**

Estimating the child’s degree of lack of problem solving.

1. Exhibits normal problem-solving abilities. Seems to be aware about his/her work style and is able to control it.

2. Shows some signs of not being able to plan a task, to change strategies when needed, nor to keep a functioning strategy. Is aware most of the time about his/her way of working but still has some difficulties to control the way of working.

3. Shows some signs of difficulties with planning, change of strategies, or maintaining strategies. Lack of self-awareness about the way of working, as well as to control it.

4. Shows difficulties with planning, change of strategies, and maintaining strategies. Lack of self-awareness about his/her way of functioning. Cannot control their behavior.

5. Great difficulties with planning, change of strategies, and maintaining strategies. Often starts all over again. Becomes frustrated by the tasks where new demands occur. Total lack of self-awareness about his/her way of working and cannot control it, even with a framework of instructions.
