# Supplementary material for: Executive Functions, Pragmatic Skills, and Mental Health in Children With Congenital Cytomegalovirus (CMV) Infection With Cochlear Implants: A Pilot Study
Source: Front Psychol. 2020 Jan 10;10:2808. doi: 10.3389/fpsyg.2019.02808 (PMC6965306; doi:10.3389/fpsyg.2019.02808)
Supplement: Supplementary file 2 [file Data_Sheet_2.docx]

**Expressive Grammar Level** (Löfkvist, 2014)

| **Level** | **Description** |
| --- | --- |
| 1 | No use of voice with intent |
| 2 | Use of voice with intent |
| 3 | Emerging CV-babbling such as /bababa/, /mamama/ |
| 4 | One-word utterances |
| 5 | Successive one-word utterances without grammatical hierarchy such as “car big» |
| 6 | Two-three word utterances |
| 7 | Multi-word sentences with atypical or incorrect grammar (articles, word order, morphology, conjunctions) |
| 8 | Typical or correct expressive grammar and sentence level |
