# Supplementary material for: Executive Functions, Pragmatic Skills, and Mental Health in Children With Congenital Cytomegalovirus (CMV) Infection With Cochlear Implants: A Pilot Study
Source: Front Psychol. 2020 Jan 10;10:2808. doi: 10.3389/fpsyg.2019.02808 (PMC6965306; doi:10.3389/fpsyg.2019.02808)
Supplement: Supplementary file 3 [file Data_Sheet_3.docx]

**Appendix 3**. Individual results on BRIEF (BRI, MI GEC) for parents (t-score); SIPS (phonological working memory and general working memory) (total raw score); CCC-2 (total score, initiative, use of context) (percentile); SDQ (Mothers, Fathers, Teachers) (total raw score).

Child BRIEF SIPS CCC-2 SDQ

(BRI; MI; GEC) (P.WM; G.WM) (IKG; Initiative; Use of context) (M; F; T)

CMV-1 47;57;53 1;11.5 2;27;1 8;11; ND

CMV-2 61;52;56 0;0 3;10;6 12;12;16

CMV-3 45;44;44 12;12 74;97;53 5;5;3

CMV-4 56;46;50 11;11 13;27;2 5;4;4

CMV-5 46;43;44 1;0 28;27;53 2;3;4

CMV-6 96;123;79 0;0 3;17;21 6;10;4

CMV-7 38;39;38 7;12 76;54;42 0;2;0

CMV-8 41;53;49 1;9 ND;ND;ND 6;7;3

CMV-9 ND;ND;ND 1;ND 19;54;33 6;ND;ND

CMV-10 68;69;70 0;9.5 29;41;12 ND;ND;ND

Cx26-11 54;52;53 18;12 95;97;93 5;0;3

Cx26-12 49;56;54 12;6 4;68;53 9;6;9

Cx26-13 40;43;41 13;15.5 93;91;83 0;2;0

Cx26-14 41;44;41 10;4.5 97;83;83 1;7;5

Cx26-15 51;59;57 3;14.5 52;68;33 3;1;10

Cx26-16 53;45;49 ND;ND 52;41;93 8;ND;3

Cx26-17 48;42;44 ND; 6.5 82;68;97 0;3;3 ______________________________________________________________________________________________

*Notes:* P.WM= phonological working memory; G.WM= general working memory; ND= not done.
